# Supplementary figures and images for: A Glyphosate-Based Herbicide Cross-Selects for Antibiotic Resistance Genes in Bacterioplankton Communities
Source: mSystems. 2022 Mar 10;7(2):e01482-21. doi: 10.1128/msystems.01482-21 (PMC9040730; doi:10.1128/msystems.01482-21)

A

MAGs composition

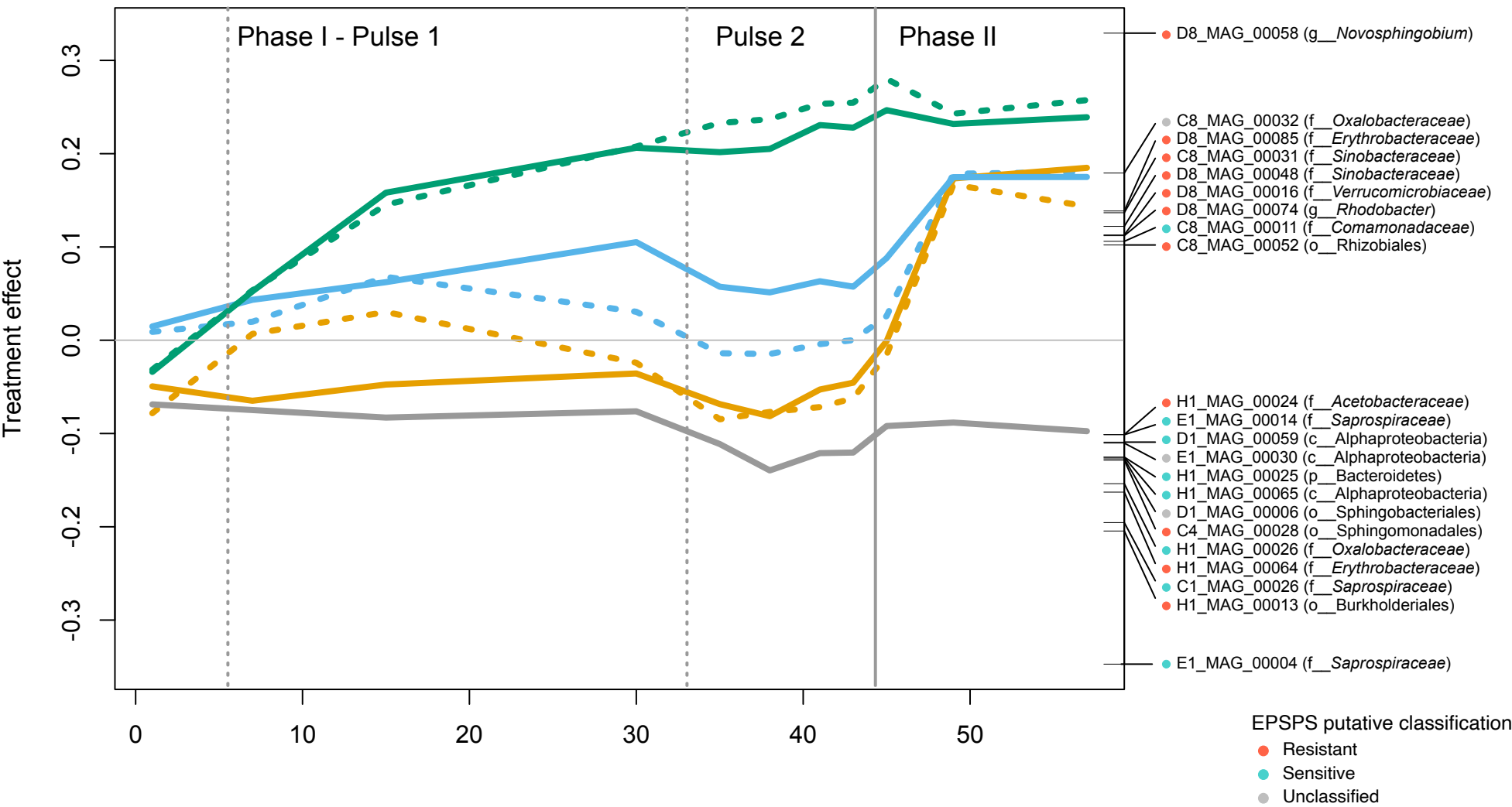

B

MAGs by phylum

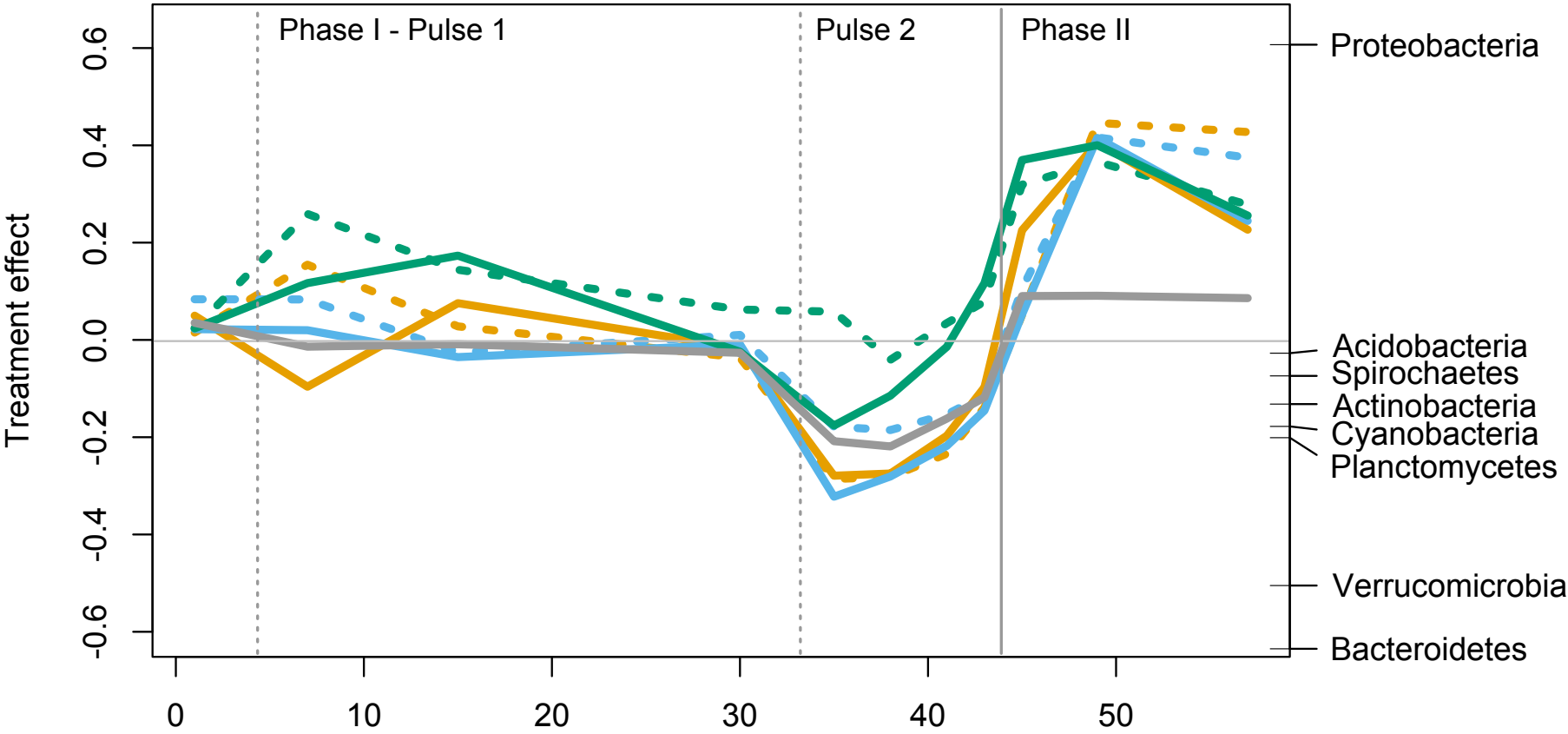

C

MAGs by class

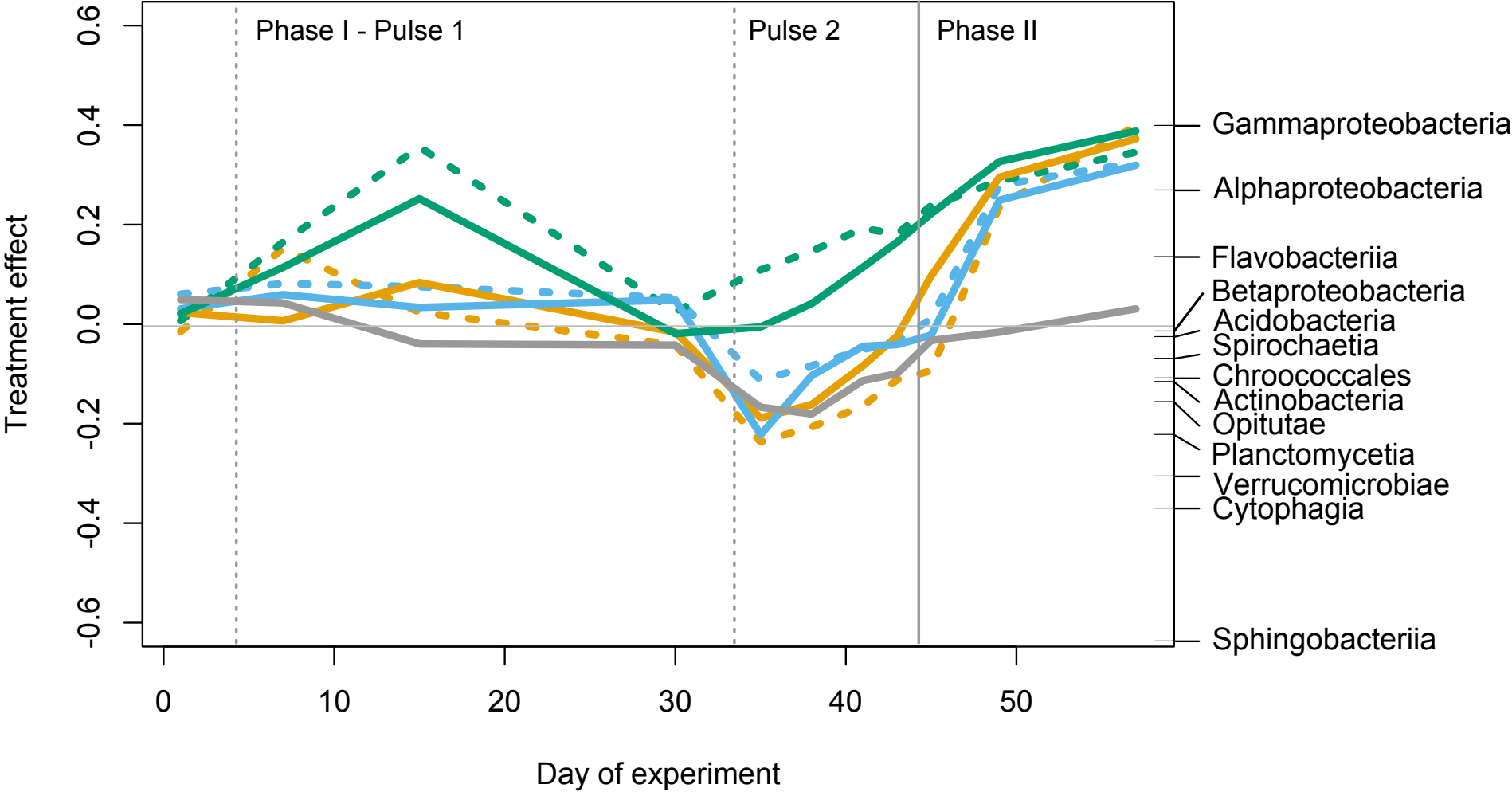

Supplement: FIG S1 [file msystems.01482-21-sf001.pdf]

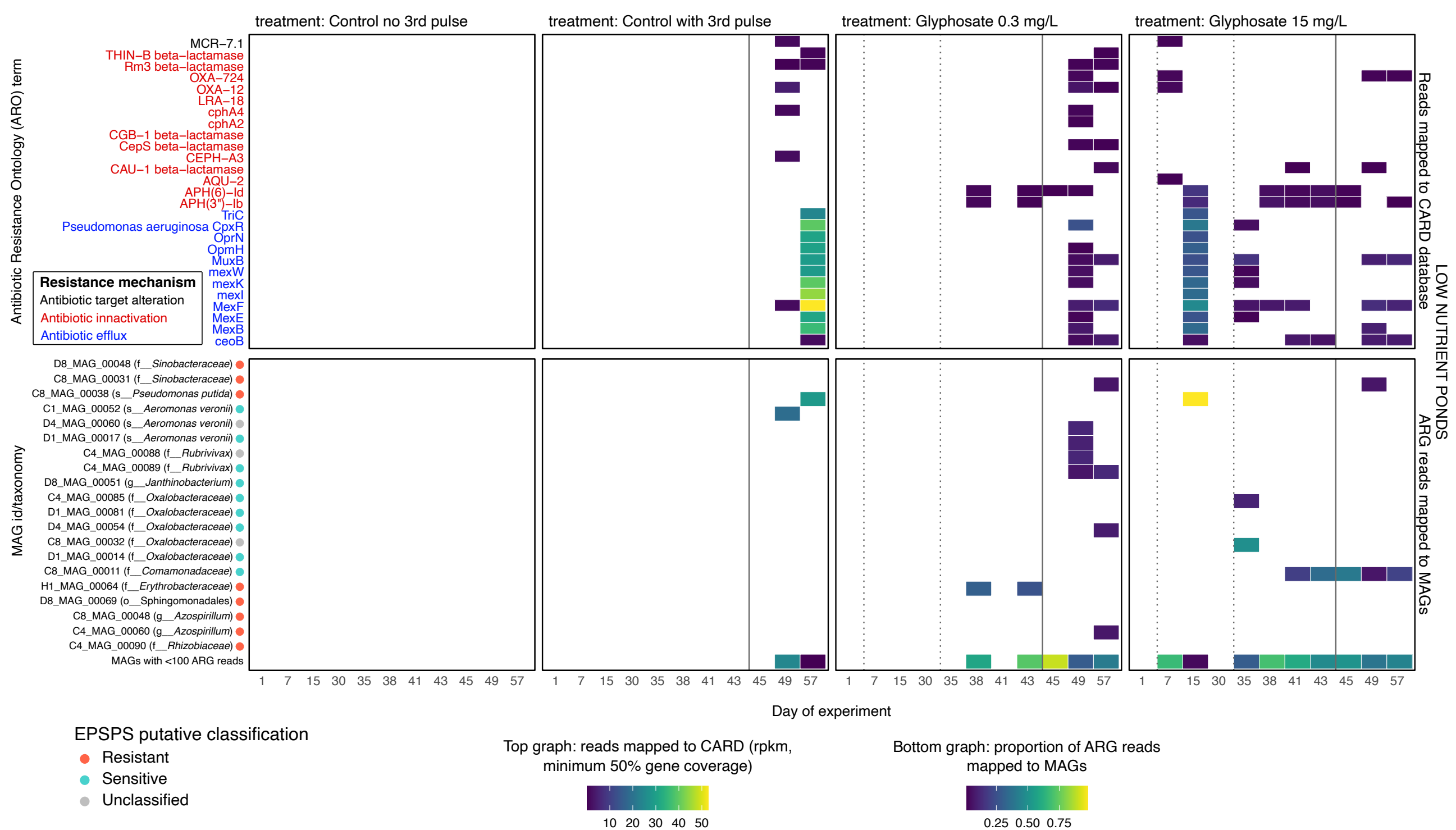

Supplement: FIG S4 [file msystems.01482-21-sf004.pdf]

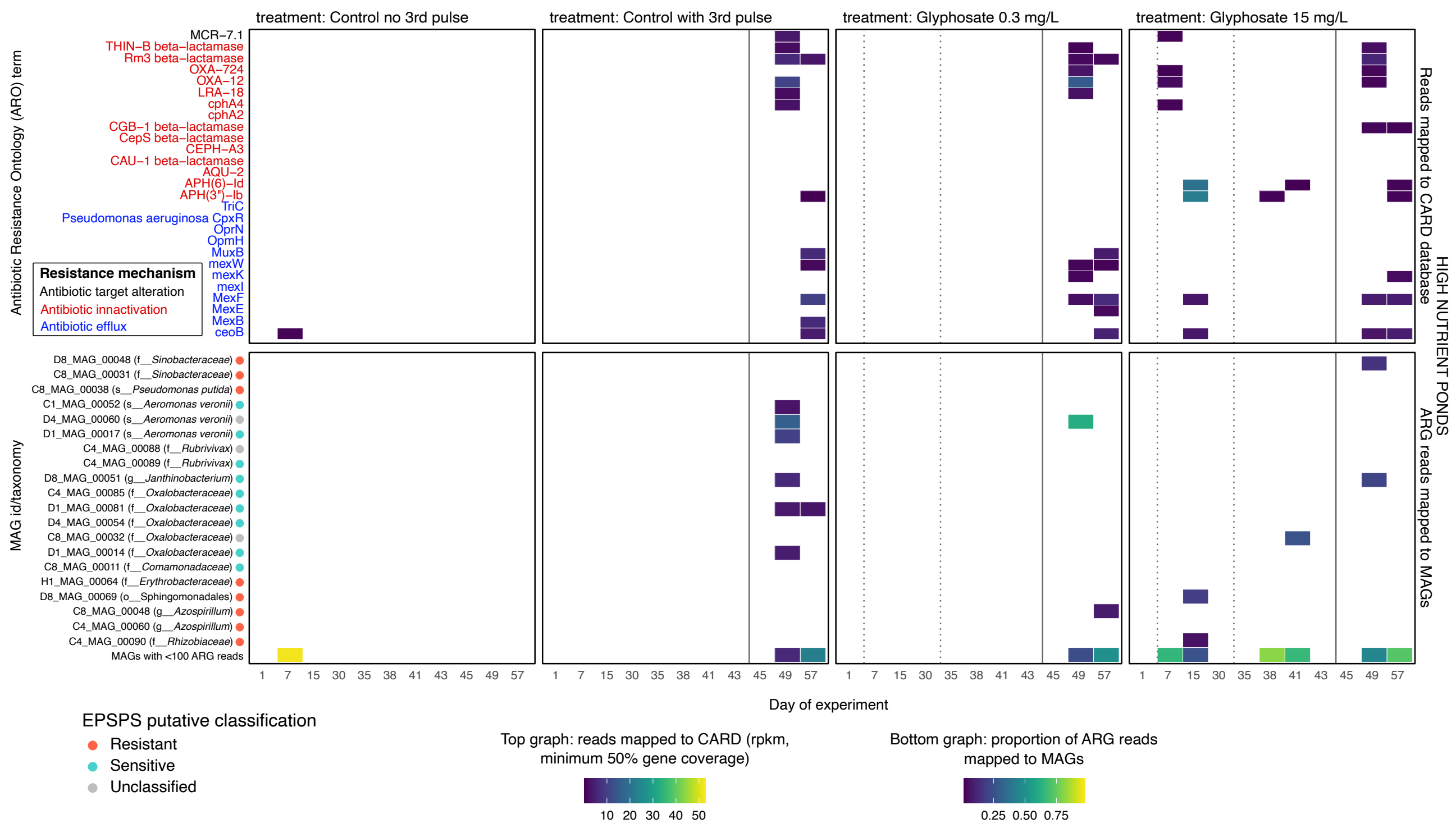

Supplement: FIG S5 [file msystems.01482-21-sf005.pdf]

**A**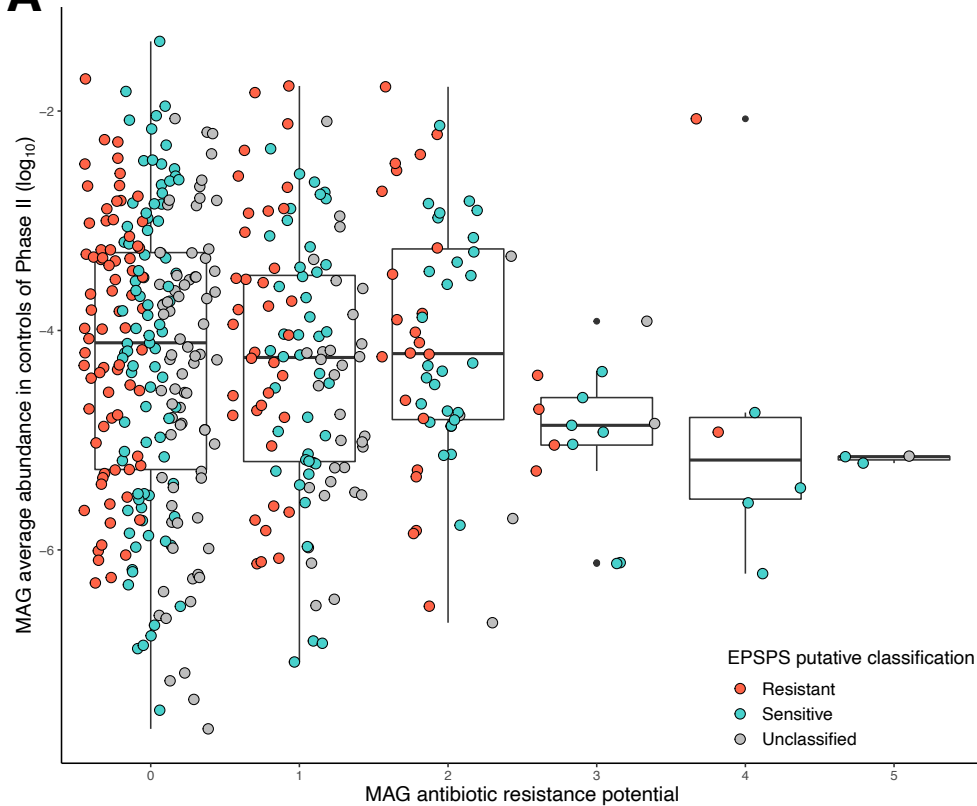**B**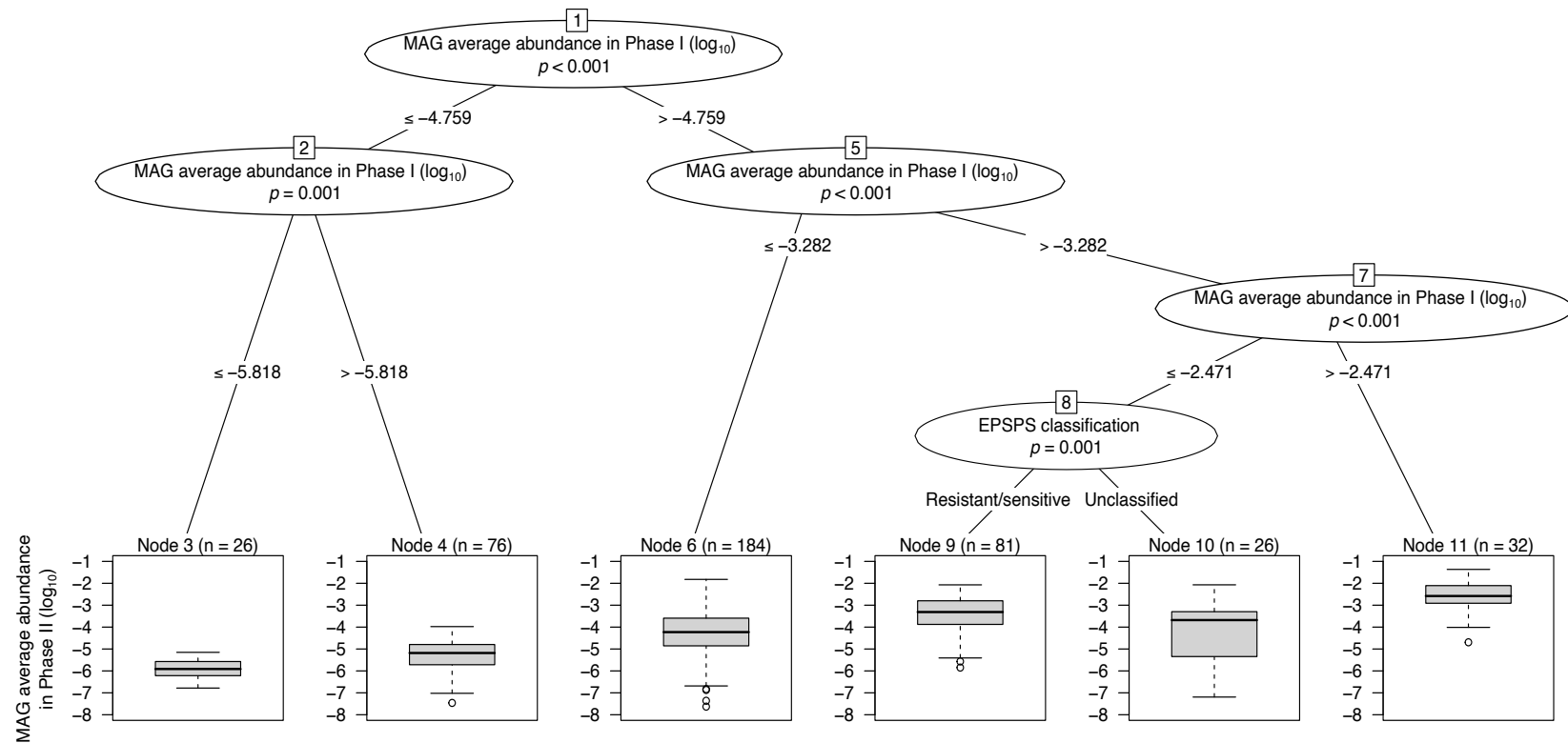

Supplement: FIG S6 [file msystems.01482-21-sf006.pdf]
