# Supplementary material for: A Glyphosate-Based Herbicide Cross-Selects for Antibiotic Resistance Genes in Bacterioplankton Communities
Source: mSystems. 2022 Mar 10;7(2):e01482-21. doi: 10.1128/msystems.01482-21 (PMC9040730; doi:10.1128/msystems.01482-21)
Supplement: FIG S2 [file msystems.01482-21-sf002.pdf]

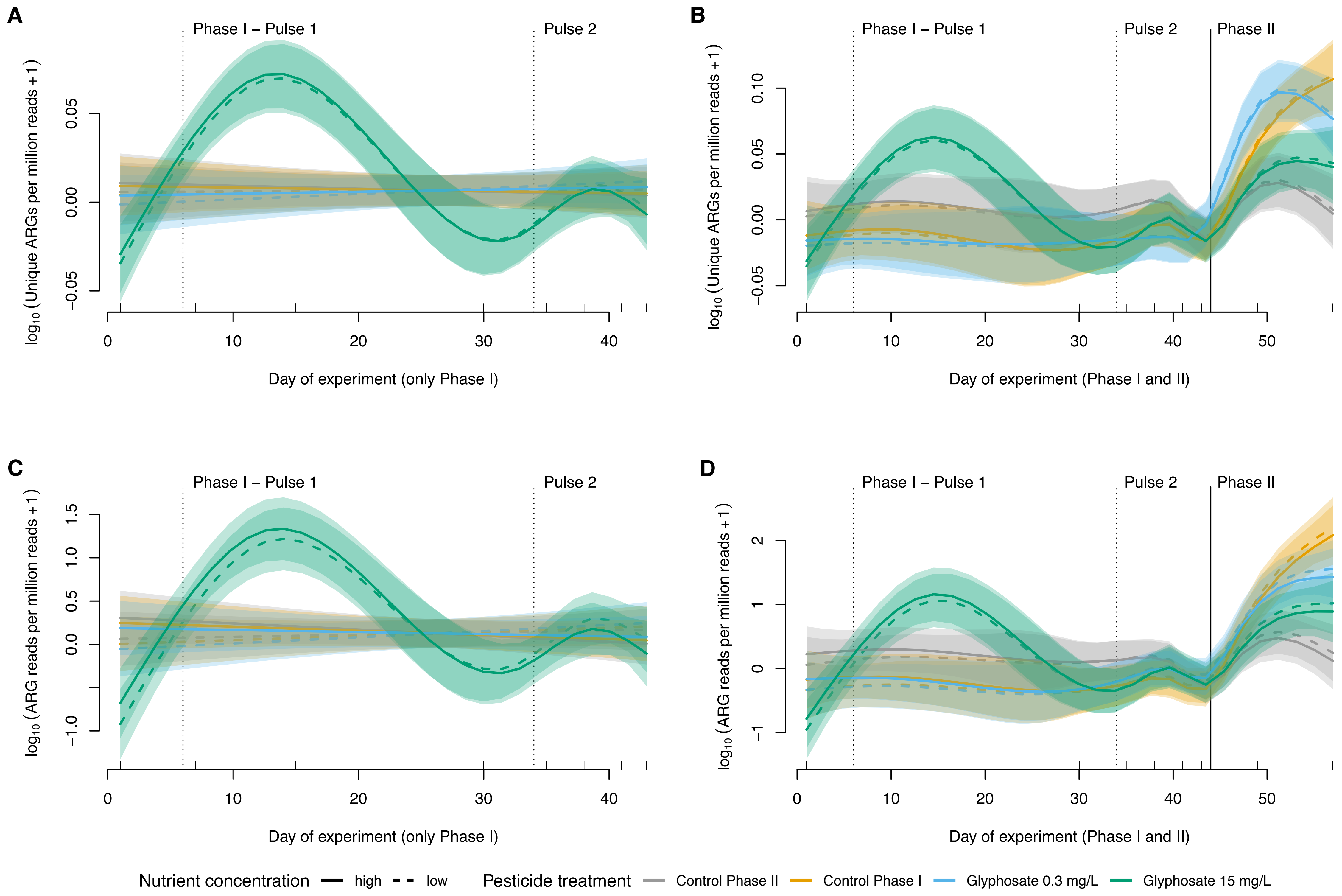

A
Phase I – Pulse 1
Pulse 2
log10(Unique ARGs per million reads + 1)
0.05
0.00
−0.05
0
10
20
30
40
Day of experiment (only Phase I)
B
Phase I – Pulse 1
Pulse 2
Phase II
log10(Unique ARGs per million reads + 1)
0.10
0.05
0.00
−0.05
0
10
20
30
40
50
Day of experiment (Phase I and II)
C
Phase I – Pulse 1
Pulse 2
log10(ARG reads per million reads + 1)
1.5
1.0
0.5
0.0
−1.0
0
10
20
30
40
Day of experiment (only Phase I)
D
Phase I – Pulse 1
Pulse 2
Phase II
log10(ARG reads per million reads + 1)
2
1
0
−1
0
10
20
30
40
50
Day of experiment (Phase I and II)
Nutrient concentration
high
low
Pesticide treatment
Control Phase II
Control Phase I
Glyphosate 0.3 mg/L
Glyphosate 15 mg/L
