# Supplementary material for: A Glyphosate-Based Herbicide Cross-Selects for Antibiotic Resistance Genes in Bacterioplankton Communities
Source: mSystems. 2022 Mar 10;7(2):e01482-21. doi: 10.1128/msystems.01482-21 (PMC9040730; doi:10.1128/msystems.01482-21)
Supplement: FIG S3 [file msystems.01482-21-sf003.pdf]

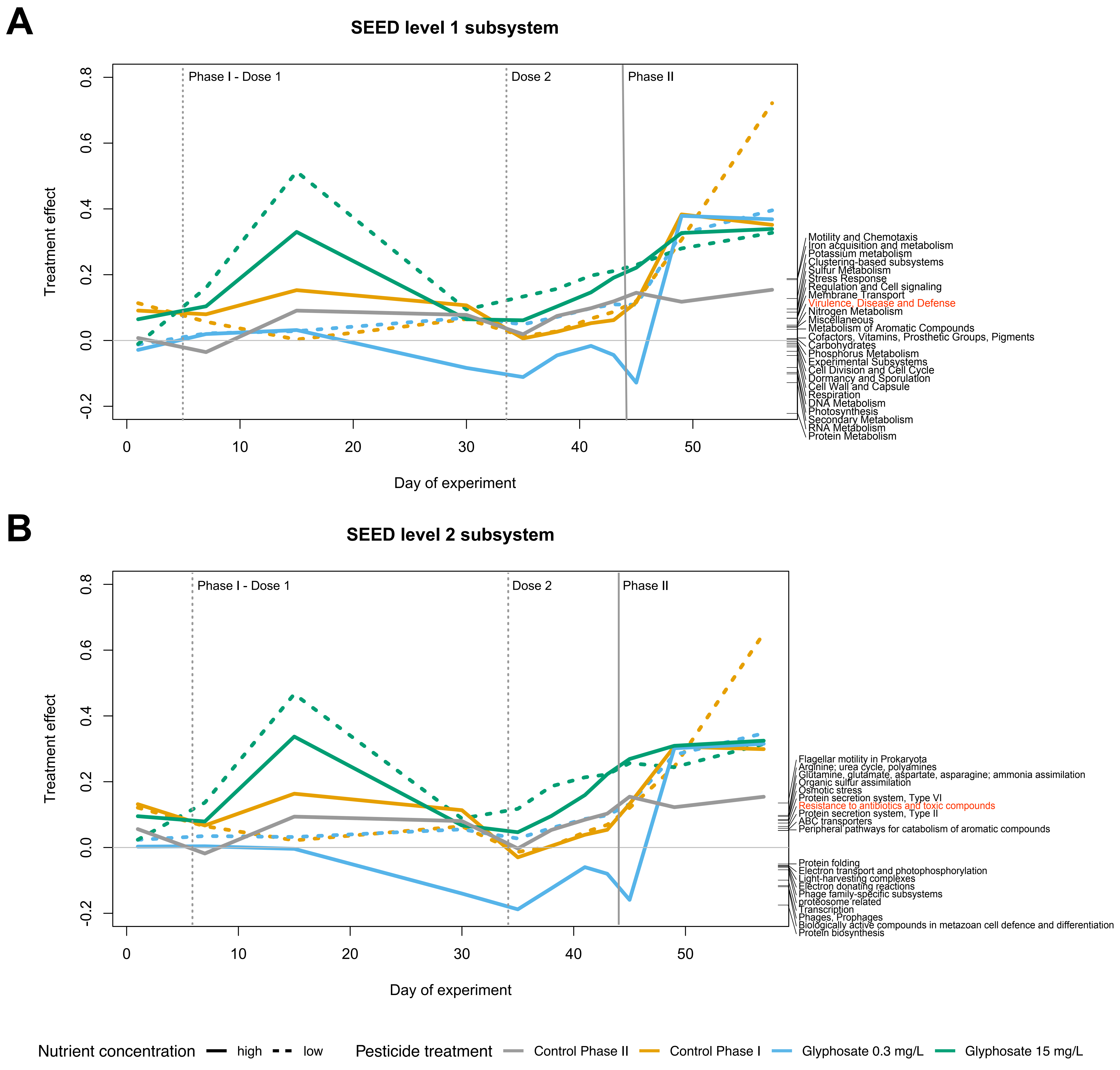
A
SEED level 1 subsystem
Phase I - Dose 1
Dose 2
Phase II
Treatment effect
Day of experiment
Motility and Chemotaxis
Iron acquisition and metabolism
Potassium metabolism
Clustering-based subsystems
Sulfur Metabolism
Stress Response
Regulation and Cell signaling
Membrane Transport
Virulence, Disease and Defense
Nitrogen Metabolism
Miscellaneous
Metabolism of Aromatic Compounds
Cofactors, Vitamins, Prosthetic Groups, Pigments
Carbohydrates
Phosphorus Metabolism
Experimental Subsystems
Cell Division and Cell Cycle
Dormancy and Sporulation
Cell Wall and Capsule
Respiration
DNA Metabolism
Photosynthesis
Secondary Metabolism
RNA Metabolism
Protein Metabolism
B
SEED level 2 subsystem
Phase I - Dose 1
Dose 2
Phase II
Treatment effect
Day of experiment
Flagellar motility in Prokaryota
Arginine; urea cycle, polyamines
Glutamine, glutamate, aspartate, asparagine; ammonia assimilation
Organic sulfur assimilation
Osmotic stress
Protein secretion system, Type VI
Resistance to antibiotics and toxic compounds
Protein secretion system, Type II
ABC transporters
Peripheral pathways for catabolism of aromatic compounds
Protein folding
Electron transport and photophosphorylation
Light-harvesting complexes
Electron donating reactions
Phage family-specific subsystems
proteosome related
Transcription
Phages, Prophages
Biologically active compounds in metazoan cell defence and differentiation
Protein biosynthesis
Nutrient concentration
high
low
Pesticide treatment
Control Phase II
Control Phase I
Glyphosate 0.3 mg/L
Glyphosate 15 mg/L
